# Update on the Role of the Non-Canonical Wnt/Planar Cell Polarity Pathway in Neural Tube Defects

**DOI:** 10.3390/cells8101198

**Published:** 2019-10-04

**Authors:** Mingqin Wang, Patrizia de Marco, Valeria Capra, Zoha Kibar

**Affiliations:** 1 Department of Neurosciences, University of Montreal and CHU Sainte Justine Research Center, Montreal, QC H3T1C5, Canada; 2 UOSD Laboratorio Neurogenetica e Neuroscienze, IRCCS Istituto Giannina Gaslini, 16147 Genoa, Italy; 3 UOC Neurochirurgia, IRCCS Istituto Giannina Gaslini, 16147 Genoa, Italy

**Keywords:** neural tube defects, planar cell polarity, convergent extension, candidate genes, animal models, human cohorts

## Abstract

Neural tube defects (NTDs), including spina bifida and anencephaly, represent the most severe and common malformations of the central nervous system affecting 0.7–3 per 1000 live births. They result from the failure of neural tube closure during the first few weeks of pregnancy. They have a complex etiology that implicate a large number of genetic and environmental factors that remain largely undetermined. Extensive studies in vertebrate models have strongly implicated the non-canonical Wnt/planar cell polarity (PCP) signaling pathway in the pathogenesis of NTDs. The defects in this pathway lead to a defective convergent extension that is a major morphogenetic process essential for neural tube elongation and subsequent closure. A large number of genetic studies in human NTDs have demonstrated an important role of PCP signaling in their etiology. However, the relative contribution of this pathway to this complex etiology awaits a better picture of the complete genetic architecture of these defects. The emergence of new genome technologies and bioinformatics pipelines, complemented with the powerful tool of animal models for variant interpretation as well as significant collaborative efforts, will help to dissect the complex genetics of NTDs. The ultimate goal is to develop better preventive and counseling strategies for families affected by these devastating conditions.

## 1. Introduction

Neural tube defects (NTDs) result from the failure of the neural tube closure during weeks 3–5 after fertilization [1,2]. Their clinical manifestation varies in type and severity depending on the site of the closure defect along the embryonic axis. They are classified into two major types: Open or closed NTDs, where the damaged nervous tissue is either exposed to the environment or covered by skin respectively. The two most common forms of open NTDs are anencephaly and myelomeningocele (MMC). The former results from the failure of the neural tube closure at the cranial region, while the latter at the spinal region. Anencephaly is characterized by an absent cranium and is invariably lethal, whereas many infants with MMC now survive but with severe and lifelong physical and developmental disabilities. A number of skin-covered (closed) NTDs are categorized clinically depending on the presence (lipomyeloschisis, lipomyelomeningocele, meningocele, and myelocystocele) or absence of a subcutaneous mass (including dermal sinus, caudal regression, and spinal segmental dysgenesis) [1].

NTDs represent the second most common group of human birth defects. Their incidence has been significantly reduced due to food fortification and maternal periconceptional supplementation of folic acid [3,4]. Their regional average prevalence per 1000 live births in 2015 varied between 0.7–1 in North America and Europe to 3.1 in Southern Asia [5]. Some NTDs are resistant to folic acid and thousands of families are still affected by these devastating conditions each year, necessitating a better understanding of their etiology and the development of novel preventive strategies [3,6]. More recently, a few pilot studies conducted in Europe have suggested a protective effect of inositol when taken periconceptionally in combination with folic acid [7,8]. Larger studies are needed to confirm this promising novel preventive strategy against NTDs.

Population and family studies indicate a complex etiology to NTDs involving environmental and genetic factors. Most NTD cases are sporadic [2,9,10]. Women who have one previous affected fetus have an empirical recurrence risk of 3% in any subsequent pregnancy and of 10% after conceiving a second NTD embryo [10]. A few families have been reported to be segregating NTDs but with a complex pattern of inheritance hindering the identification of the underlying genetic variants [9,11]. NTDs in humans are hypothesized to follow a multifactorial threshold model where many genetic variants, alone or in interaction with many environmental factors, modulate the incidence and severity of the disease. Heritability in NTDs is estimated to be 60% with multiple susceptibility genes involved [12]. However, the identity and relative contribution of such genes to NTDs remain unknown.

This article reviews the role of the non-canonical Wnt/planar cell polarity (PCP) genes in NTDs’ pathogenesis. The process of neural tube formation and how defects in this process lead to the various types of NTDs is first briefly described. Then, the components and readouts of PCP signaling during development are described. The focus is placed on the process of convergent extension that is mediated by PCP signaling and that is the major force underlying the elongation and narrowing of the neural tube. This is followed by a summary of the undebatable evidence of an important role of defective PCP signaling in the causation of NTDs in animal models and an update on the findings from genetic studies of this pathway in human NTDs. Finally, the contribution of this pathway is discussed in the larger context of the genetic architecture of NTDs and the challenges faced by researchers in their quest for dissecting the complex etiology of these debilitating birth defects and their ultimate prevention.

## 2. Neural Tube Formation: A Rapid, Multi-Step and Complex Process

The neural tube is the precursor of the brain and spinal cord. Its formation is a dynamic and rapid process that occurs in 2 phases: Primary neurulation that leads to formation of the brain and most of the spinal cord during weeks 3 and 4 of pregnancy; secondary neurulation that occurs in the lower sacral and coccygeal regions during week 5 of pregnancy [1].

**Figure 1:**
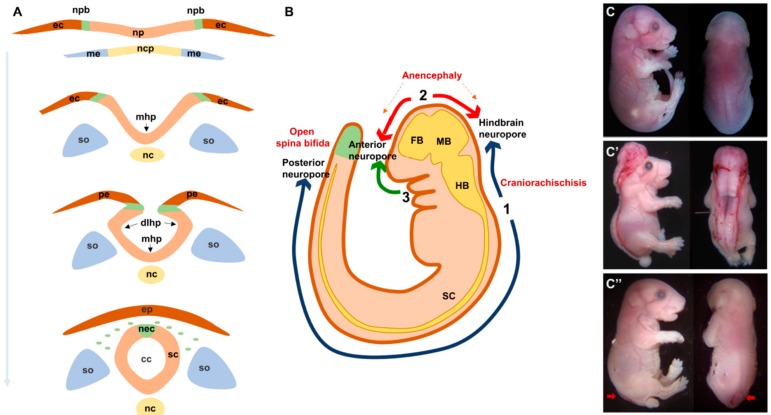
Normal and abnormal neural tube formation. (**A**) Major steps of neural tube formation. The neural plate overlying the notochord elevates to form the neural folds around the midline, bends at the midline (mhp) and dorsolateral sites (dlhp) then fuses at the opposing tips of the neural folds and separates from the overlying epidermis. (**B**). Initiation sites of neural tube closure 1–3 in a mouse embryo. Defects in closure 1 and 2 lead to craniorachischisis and anencephaly respectively. Defects in closure at the caudal end of site 1 lead to open spina bifida. (**C**). Lateral and dorsal views of E18.5 embryos showing craniorachischisis (**C′**) and open spina bifida (**C″**, indicated by red arrows) as compared to wild type (**C**). cc, central canal; dlhp, dorsolateral hinge point; ec, ectoderm; ep, epidermis; fb, forebrain; hb, hindbrain; mb, midbrain; mhp, median hinge point; me, mesoderm; nc, notochord; ncp, notochordal plate; nec, neural crest; np, neural plate; npb, neural plate border; pe, presumptive epidermis; sc, spinal cord; so, somites.

Primary neurulation starts with a flat sheet of specialized dorsal ectodermal cells, the neural plate, which first undergoes extensive shaping and then elevates to form bilateral neural folds that bends to face one another across the midline, fuse at their tips, and separate from the overlying non-neural ectoderm (Figure 1A). The dynamic development of the neural tube is also mediated by the signals and biomechanical forces from the surrounding tissues [13,14].

Shaping of the neural plate involves its convergence towards the midline and extension along the anterior-posterior axis. This morphogenetic process of convergent extension (CE) is discussed in details below. As CE is progressing, the neural plate elevates towards the dorsal midline leading to the formation of bilateral neural folds. This is followed by formation of 3 bending or hinge points that facilitate the apposition of the neural folds across the midline: one median hinge point (MHP) at the midline overlying the notochord and two dorsolateral hinge points (DLHP) [13,14] (Figure 1A). The pattern of HP formation along the neural tube is influenced by factors secreted by the notochord, mainly Sonic Hedgehog, and by the surface ectoderm, mainly Bone Morphogenic Proteins [15,16]. The major morphogenetic process that occurs during HP formation is apical constriction whereby cells convert their shape from cuboidal to wedge-like due to a decrease in their apical area and that is mediated mainly by an actin-binding protein called Shroom3 [17,18,19]. The last step of NT closure is fusion of the opposing tips of the neural folds and the separation of the closed NT from the overlying non-neural ectoderm which later develops into the epidermis (Figure 1A). This step involves significant tissue remodeling and dynamic behaviors including the formation of cellular bridges, ruffles, filopodia and lamellipodia [20]. The molecular mechanisms underlying this step remain largely unknown but a few studies implicated members of the protease activated receptors signaling (Par1 and Par2) [21], the grainyhead-like transcription factors (Grhl2) [22], the Rho family of GTPases (Rac1 and Cdc42) [23] and EphrinA-EphA interactions [20,24].

Neural tube closure is initiated at specific sites along the embryonic axis and proceeds in a zipper-like fashion rostrally and/or caudally (Figure 1B). These sites have been well defined in mouse models and are still poorly defined in humans. In mice, closure 1 occurs at the hindbrain/cervical brain boundary, closure 2 at the forebrain/midbrain boundary and closure 3 at the rostral end of the future forebrain. Closures 1 and 2 proceed bidirectionally while closure 3 proceeds only caudally. These multiple closure sites create three neuropores: The anterior and hindbrain neuropores in the cranial region and the posterior neuropore in the low spinal region [2,9]. In humans, gross and histological examination of fetuses support a multisite closure model. However, inconsistencies in the number of sites varying between 2 and 5 have been reported [25,26,27].

Secondary neurulation is a canalisation process where a group of self-renewing stem cells in the caudal tailbud condense and undergo canalisation, converting the solid neural precursor into a hollow secondary neural tube that becomes continuous with the primary NT [13,14]. The cellular and molecular mechanisms underlying secondary neurulation remain largely unknown. Fate mapping studies in chick embryos suggested similar cellular or molecular mechanisms to primary neurulation [28].

## 3. Neural Tube Defects: What Could Go Wrong?

Any lesion at any of the mechanisms described above could lead to NTDs. In mouse models, the mutations in at least 250 genes were found to be responsible for the failure of NT closure [29]. The majority of these mutations follow a simple Mendelian mode of inheritance with high penetrance, as opposed to the complex pattern inheritance observed in humans. The type and severity of the resulting NTD depend on which closure site was affected. In mouse models, the failure of closure 1 leads to a rare and very severe form of NTDs called craniorachischisis (CRS) characterized by an open neural tube throughout the spinal region. Incomplete closure 1 at the caudal side causes open spina bifida (Figure 1C). The failure of closure 2 leads to anencephaly and that of closure 3 causes a split face and usually a forebrain anencephaly. The defects in secondary neurulation are thought to cause the milder forms of closed NTDs and often involves tethering of the spinal cord along with lipoma [9,14].

## 4. PCP Signaling: A Non-Canonical Branch of Wnt Signaling with Unresolved Mysteries

### 4.1. Non-Canonical Wnt/PCP Signaling: From Flies to Vertebrates

**Figure 2:**
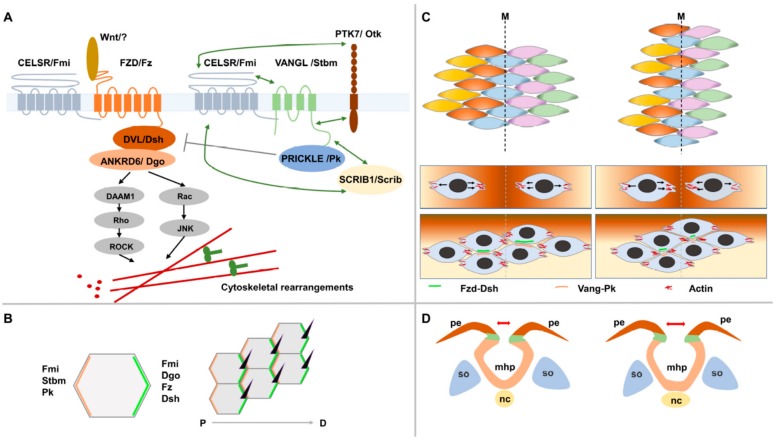
The non-canonical Wnt/planar cell polarity signaling pathway (PCP). (**A**) A simplified diagram of the PCP signaling pathway showing its core components, its downstream effectors and two of its mediators Ptk7/Otk and Scribble1/Scrib. Nomenclature for core proteins is indicated in vertebrates and Drosophila (separated by/). Genetic interactions are indicated by double arrows. (**B**) An example of a PCP-regulated process in the wing blade of Drosophila. Asymmetric localization of PCP protein complexes at the proximal (Vang-Pk) and distal (Fz-Dsh) sides of adjacent cells mediates the distal positioning and orientation of a single trichome in each cell of the wing epithelium. (**C**) The process of convergent extension. Cells elongate mediolaterally, move and intercalate with neighboring cells resulting in convergence toward the midline and extension along the anteroposterior axis. During this process, PK and VANGL localize anteriorly whereas FZD and DVL localize posteriorly. (**D**) A simplified diagram showing a widened neural plate because of a defective convergent extension (CE) of midline neuroepithelial cells. The neural folds will be far apart and will fail to fuse leading to a neural tube defect.

The first Wnt-1 gene was discovered in 1982 and since then, an explosion of studies has been conducted to dissect the role of this gene along with other Wnt members in signaling in development and disease [30,31,32]. This long-studied branch of Wnt signaling is referred to as canonical and is regulated by the intracellular levels of ß catenin. It has a crucial role in mediating the cell proliferation and cell fate during development and is deregulated in many types of cancer [32,33]. Canonical Wnt signaling shares upstream effectors with another less well-studied and more recently discovered branch called the non-canonical PCP pathway [34]. PCP is β-catenin independent and mediates tissue polarity that describes the collective cell polarity within the plane of an epithelium and that is usually perpendicular to the apicobasal polarity within the cell [34,35]. It was first described in the fly where it mediates the polarity of highly organized structures including the distal orientation of wing trichomes (hair cells) and the complex organization of the ommatidia (eye units) in the adult eye [36,37]. Genetic studies of mutants affecting these structures have identified a group of six core PCP genes: *Vang gogh*/*Strabsimus* (*Vang*/*Stbm*), *Frizzled* (*Fz*), *Dishevelled* (*Dsh*), *Flamingo* (*Fmi*), *Prickle* (*Pk*) and *Diego* (*Dg*) (Figure 2A). PCP in the developing wing is established through an asymmetric localization of two mutually exclusive signaling complexes along its proximal-distal axis (Figure 2B). Upon binding of PCP-specific Wnt ligands to the transmembrane protein Fz receptor, the latter recruits the cytosolic proteins Dsh and Dgo distally while the transmembrane protein Vang/Stbm recruits the cytosolic protein Pk and localizes it proximally. The atypical cadherin Fmi is localized to both proximal and distal membranes and promotes the assembly of these complexes and the propagation of PCP asymmetry throughout the tissue (Figure 2B). In the fly, it was shown that PCP signaling directly impacts the cytoskeleton by controlling the actomyosin machinery via Rho GTPases and Rho Kinase and thereby inducing the observed morphological and cell behaviour changes (Figure 2A) [35,36,37].

The studies of vertebrate models have demonstrated a high level of evolutionary conservation at PCP core genes (Figure 2A) and have identified a new set of PCP genes including those coding for PCP vertebrate-specific Wnt ligands (Wnt 5A, 5B and 11) and Wnt co-receptors (Ryk, Ror2 and Ptk7) [37,38,39,40]. These studies also discovered a new role of the apicobasal polarity gene *Scribble1* (*Scrib1*) in PCP signaling [41]. PCP signaling in vertebrates mediates planar polarity and collective cell movements in a large number of developmental processes and in some pathogenic states. Among these processes are the convergent extension during gastrulation and neural tube formation, axonal guidance, neuronal polarity, eyelid closure, hair bundle orientation in inner ear sensory cells, hair follicle orientation in the skin, epidermal wound repair, and branching morphogenesis in lung and kidney. The defects in PCP signaling in vertebrates result in a range of developmental anomalies and diseases including NTDs, polycystic kidneys, and congenital heart diseases [42,43,44,45,46]. Recent studies have discovered an important role of this pathway in promoting directed cell migration during the invasion and metastasis of malignant cells [47,48]. The underlying molecular mechanisms of PCP signaling in health and diseases remain a mystery despite extensive studies conducted in animal models.

### 4.2. PCP and CE during Neural Tube Formation: Strong Evidence from Animal Models

The first process that was shown to be mediated by the non-canonical Wnt/PCP signaling in vertebrates was the convergent extension during gastrulation and neurulation in Xenopus and zebrafish. CE involves two major types of cell movements: Collective cell migration followed by cell intercalations. Neuroepithelial cells produce mediolaterally-oriented protrusions that enable them to move directionally in a manner similar to the leading edge of a migrating cell. Next, mediolaterally-oriented junctions undergo actomyosin contractions, thereby driving cell intercalations (Figure 2C) [35,49,50,51]. Extensive studies of these models have established a similar molecular basis of PCP signaling to that described in Drosophila from the first step of the asymmetric localisation of the transmembrane complexes to the downstream effectors of actin regulators including RhoA, Rho kinase, the formin Daam1 and the Rho GTPase Cdc42 [35]. In vertebrate cells undergoing CE, it was shown that PK and VANGL localize anteriorly, whereas DVL localizes posteriorly [35] (Figure 2C). The knockdown or overexpression of PCP genes in Xenopus and zebrafish caused CE defects, manifested by a shortened body axis and an open neural tube (in Xenopus) [35,52,53,54]. In mammals, the defects in PCP signaling impair CE and neural tube formation [2,9,55]. In fact, the first mouse model to implicate PCP signaling in NTDs was the Looptail (Lp) mouse that suffers from the severe form of CRS. The traditional linkage and positional cloning studies have identified the core PCP gene *Vangl2* as the gene defective in Lp [56,57]. It was demonstrated in this mutant that disrupting non-canonical Wnt/PCP core genes caused a defective CE of midline neuroepithelial cells that led to a widened neural plate and far apart neural folds that failed to fuse (Figure 2D) [58]. The subsequent studies of other single or double mouse mutants at other core PCP genes, *Celsr1* (orthologue of *Fmi*) [59], *Fz3*/*Fz6* [60] and *Dvl1*/*Dvl2* [61], and at vertebrate PCP genes, *Ptk7* [38] and *Scrib1* [41] have further confirmed the role this pathway in NTDs’ pathogenesis. CRS is the hallmark phenotype of a defective PCP signaling in mice. However, it was demonstrated that double-mutant embryos at PCP genes (*Vangl2*, *Scrib1*, *Celsr1* and *Ptk7*) generated a range of open NTDs including anencephaly and open spina bifida [38,62].

### 4.3. PCP: Molecular Crosstalks to Other Processes and Pathways during Neurulation

PCP signaling does not act alone during neurulation. It forms part of an intricate and finely regulated network of processes and signaling pathways that together ensure proper neurulation. Among these processes are apicobasal polarity (ABP) [41,63], apical constriction [64], oriented cell divisions [43,44], ciliogenesis [35,65], and the canonical Wnt signaling pathway [34]. The molecular cross-talks between PCP and each of ABP and the Wnt canonical signaling that have been associated with NTDs in mouse mutants and human cohorts are briefly discussed.

*PCP and ABP*: ABP is essential for normal epithelial cell shape and function and is generated and maintained by antagonistic interactions between the apical aPkc–Par6–Par3 and Crumbs–Pals1–Patj complexes and the basolateral proteins Scribble, Lgl and Dlg [66]. The defects in ABP affect tissue integrity and structure and have been linked to many types of cancer [67]. *Scrib1* was the first gene to link ABP to PCP signaling. In mice, mutations in *Scrib1* cause CRS and genetically interact with the core PCP gene *Vangl2* to mediate the orientation of ear sensory cells, CE and neural tube formation [41,63]. Histological studies of the developing neural tube in these mutants and of cultured epithelial cells following partial knockdown of *Scrib1* showed an abnormal localization of Vangl2 and of the apical protein Par-3 [68]. It was hypothesized that mutations in *Scrib1* cause or predispose to NTDs through its effect on Par-3 and Vangl2 localization and most likely independently of ABP [68]. Additional studies have confirmed a role for Scrib during neural tube morphogenesis in zebrafish [69] and in Drosophila eye and wing development as part of PCP signaling [70]. The details of genetic studies of *SCRIB1* in human NTDs are described below.

*PCP and the canonical Wnt/β-catenin pathway*: Several studies have identified a group of molecular switches that act on both Wnt/β-catenin and Wnt/PCP pathways simultaneously in the neural tube closure [71]. These mediators include Lrp6 and Ptk7. *Lrp6* acts as a Wnt co-receptor and is crucial for activation of Wnt/β-catenin canonical pathway while simultaneously inhibiting the non-canonical pathway [72,73]. *Lrp6* downregulation in Xenopus embryos disrupts CE during gastrulation [74] and knockout in mice causes exencephaly, spina bifida, absent tail, and limb deformities [75,76]. Interestingly, PCP defects in both Xenopus and mouse embryos lacking Lrp6 function were rescued by the deletion of the non-canonical ligand Wnt5a, indicating that these phenotypes resulted from non-canonical Wnt gain-of-function [72,73,74]. Ptk7 activates the Wnt/PCP pathway while simultaneously inhibiting the Wnt/β-catenin canonical pathway [77]. *Ptk7* mouse mutants suffer from CRS and genetically interact with *Vangl2* [38]. In frog and zebrafish models, morpholino oligonucleotides (MO) induced knockdown of *Ptk7* as well as its overexpression lead to similar CE defects manifested mainly by a shortened body axis and widened somites [77,78]. Depending on the context, Ptk7 is also able to positively regulate Wnt signaling in the posterior neural plate in Xenopus through the interaction with ß catenin. The depletion of *Ptk7* in these cells inhibited the induction of direct targets of canonical Wnt signaling resulting in a loss of posterior neural cell fates and preventing later neural CE [79,80]. The details of genetic studies of *LRP6* and *PTK7* in human NTDs as mediators of PCP signaling are described below.

## 5. PCP Signaling Genes in Human NTDs: What do We Know So Far?

### 5.1. Genetic Studies of PCP Signaling in Human NTDs

The strong candidacy of PCP genes in NTDs’ pathogenesis as deduced from animal models has provided a solid rationale for investigating their orthologues in human NTDs. The first genetic study of PCP signaling in humans NTDs implicated orthologues of *Vangl2* mutated in Looptail. In that study, the authors analyzed *VANGL2* and its paralogue *VANGL1* in a cohort of 137 Italian patients with nonsyndromic spinal dysraphisms and 7 fetuses with CRS [81]. Surprisingly, they did not detect any nonsynonymous variant in *VANGL2*. On the other hand, they identified in *VANGL1* three variants detected in a heterozygous state, p.Val239Ile, p.Arg274Gln, in two familial NTD cases and p.Met328Thr in one sporadic case. The p.Val239Ile was a de novo mutation that abrogated the interaction between VANGL1 and all three Dvl proteins [81]. However, this study was published more than a decade ago at a time where the majority of researchers in this field adopted a candidate gene approach to dissect the complex genetics of NTDs. It had a high impact in the field of NTD genetics as it was the first genetic and functional study that demonstrated a role of PCP signaling in human NTDs [81]. Since then, a large number of studies have been conducted on the role of PCP core genes and mediators in NTDs’ pathogenesis making it the most extensively studied pathway in human NTDs [81,82,83,84,85,86,87,88,89,90,91,92,93,94,95,96,97,98,99,100,101,102]. The majority of these studies included genetic as well as functional data in cell cultures and zebrafish models. Table 1 summarizes the major positive findings from these studies. It lists rare PCP variants that were associated with NTDs and that were predicted to be pathogenic and/or functionally validated. This table is an underestimate of the contribution of PCP signaling to NTDs. It does not include rare variants that were predicted to be benign and/or were not functionally validated and/or synonymous that could affect gene splicing and/or regulatory that could represent novel PCP mutations in human NTDs. Most of these studies focused on coding rare variants present in patients and might have missed other rare damaging variants because of the strategy of excluding variants present in as few as one control. Only a few studies did not identify NTD-associated variants of each of *DVL3*, *FZD3*, *PTK7*, *VANGL1*, *VANGL2* and *PK1*, in the cohorts analyzed and were not included in this table [82,89,90,91,102]. Only a few studies assessed the role of common variants of PCP genes (MAF > 1%) in NTDs and the majority did not find a significant association between these variants and the NTDs’ phenotype [82,83,86,87]. Only one study has detected a significant association between the common SNP rs4839469 in *VANGL1* and NTDs [103]. 

In mice, single mutants of PCP genes suffer mainly from the severe CRS and rarely exhibit open spina bifida [38,41,56,57,59,60,61]. CRS is an extremely rare type of NTDs in humans hindering the availability of large cohorts for genetic studies. Assuming no overlap in the cohorts included in Table 1, 8 PCP genes (*VANGL1-2*, *CELSR1-3*, *DVL3*, *SCRIB1* and *PTK7*) have been re-sequenced in a couple of hundred of CRS cases and no obvious loss of function (LoF) variant was identified in these genes in the majority of CRS cases analyzed. Interestingly, the double mutants of PCP genes in mice exhibit a wide range of open NTDs including anencephaly and open spina bifida, providing a strong rationale for a comprehensive analysis of this pathway in other types of open NTDs in humans [38,62]. A large number of studies have been conducted on the role of PCP signaling in open NTDs with a focus on the most common type that is MMC. As shown in Table 1, potentially pathogenic rare variants were identified in each of the 14 PCP genes in a small fraction of open NTD patients. As in the case for CRS, the majority of these variants were missense with no obvious LoF effect. Further shown in Table 1, a few PCP studies have also included closed NTDs and obtained similar types of genetic variants, suggesting a shared role for this pathway in the pathogenesis of open and closed forms of NTDs.

The majority of NTD cases are sporadic. A few families of Italian origin with multiple individuals affected by open or closed NTDs were available for genetic studies of PCP. The potentially damaging PCP variants identified in these families did not segregate with the NTDs’ phenotype, consistent with a complex pattern of inheritance for NTDs [68,83,85]. In both sporadic and familial cases of NTDs and when parents’ DNA was available, the majority of PCP variants were inherited from apparently healthy parents indicating low penetrance. Many PCP variants initially reported to be found only in a single family appeared later in the ExAC and gnomAD databases at a very low frequency. This should not be surprising as the vast majority of unaffected individuals are expected to carry one or more risk alleles and even large effect alleles are subject to background modification and incomplete penetrance.

All variants identified in PCP genes in NTDs were heterozygous in contrast to the recessive mode of action of mutations identified in their orthologues in mouse mutants. The most plausible interpretation for this finding is that these variants need to interact with other genetic variants and possibly environmental factors to modulate the incidence and severity of the NTD phenotype. Hence, NTDs might be explained by the inheritance of different combinations of mutant alleles in different individuals. Researchers have tried to investigate this hypothesis by the identification of NTD patients who are double or compound heterozygotes for damaging variants of PCP genes and by assessing the genetic burden of PCP in human NTDs. NTD patients who were double heterozygous for potentially pathogenic PCP mutations were identified in many studies but were not further investigated for a combined functional effect on PCP signaling [87,91,95,97,98,101]. One recent study used targeted next generation sequencing (NGS) of 30 PCP genes in 184 Chinese NTD cases followed by a replication of findings in *CELSR1* in an independent cohort of 292 American patients [100]. This study identified double damaging variants in *CELSR* genes and other PCP genes in 3.3% of their cohort and demonstrated a combined effect of two heterozygous variants of *CELSR2* and *DVL3* in down regulation of PCP signaling in vitro. The same study demonstrated a significant enrichment of LoF variants in *CELSR1* in NTDs when compared to the frequency reported in the ExAC database. Other studies have demonstrated a significant enrichment of predicted damaging variants in NTDs compared to controls in 3 other PCP genes: *VANGL1* [97], *FZD6* [89] and *PTK7* [102].

The functional validation of missense variants identified in NTD patients in core PCP genes focused mainly on protein-protein interactions and on protein localization that are essential for proper PCP signaling. For example, variants of *VANGL* genes affected the interactions with DVL members and vice versa [81,84,100] and variants of *CELSR1* affected its membrane localization and its recruitment of Vangl2 to the cell membrane [90,94]. Other validation assays included the zebrafish model where abnormal PCP signaling led to a defective CE manifested by a shortened body axis and widened somites. This easily measured phenotype was used as a readout for testing any potential pathogenic effect of PCP variants on the process of CE. For example, two variants of *VANGL1* failed to induce CE defects when overexpressed and to rescue MO-induced CE defects and they were hypothesized to be hypomorphic or LoF [104]. Three variants of *PK1* were hypothesized to be hypermorphic in an overexpression assay and one of these variants antagonized the CE phenotype induced by the overexpression of the wild-type *PK1* in a dominant fashion [85]. On the other hand, the functional validation of variants detected in PCP mediators including *LRP6* and *ANKRD6* (that codes for DIVERSIN) mainly focused on their abilities to act antagonistically on both Wnt pathways. Interestingly, the variants of each of these 2 genes were shown to lose this antagonistic effect indicating the importance of a tightly regulated balance between Wnt pathways during neural tube formation [93,95,96]. The variants identified in *SCRIB1* followed an unexpected finding of its effect on Par3 and Vangl2 localization in the neural tube in mouse mutants. Two variants of *SCRIB1* failed to rescue the localization defect of Par-3 and Vangl1 caused by knockdown of *SCRIB1* in MDCK cells [68].

### 5.2. Are PCP Genes Major Culprits and/or Accomplices in the Complex Etiology of Human NTDs?

Following the summarized findings of genetic studies of PCP signaling in human NTDs described above, can the relative contribution of PCP genes to NTD pathogenesis be predicted with any accuracy? Our current state of knowledge of the genetic architecture of these defects, similar to many other medically important complex diseases, is still too limited to answer this question. As mentioned above, NTDs have long been hypothesized to follow a multifactorial threshold model where a combination of a large number of genetic variants and environmental factors are needed to reach this threshold and develop a phenotype. The genetic component remains poorly defined. As severe forms of NTDs are deleterious to fitness, it is reasonable to hypothesize that predisposing genetic variants should be selected against and should therefore be rare. However, is genetic liability to severe NTDs attributed to an accumulation of rare alleles with large effects and/or rare alleles that all have minor effects? One WES study in 43 sporadic cases affected with MMC or anencephaly demonstrated an important role of LoF de novo mutations (DNMs) in the development of these severe forms of NTDs [106]. DNMs are more deleterious, on average, than inherited mutations and are represented as “single mutations with large effects” that play a major role in many rare and common forms of developmental diseases [107,108]. Genetic studies of PCP signaling described above demonstrated an enrichment of damaging variants in PCP genes in severe NTDs. While most of these mutations were missense, heterozygous and incompletely penetrant, it is still possible that they can exert a large effect in NTDs’ pathogenesis. PCP signaling is extremely important for tissue formation and therefore it is reasonable to suggest that obvious LoF variants of this pathway are not compatible with life and can escape detection. On the other hand, a recent whole genome sequencing study conducted in three different NTD cohorts (Han Chinese, Caucasian USA, and Middle Eastern/Qatar) have proposed an omnigenic model for genetic liability to NTDs that is determined by an accumulation of singleton LoF variants of many genes, all with minor effects. They did not detect any significant enrichment of these variants in any biological pathway. Interestingly, they showed that the accumulation of nine of these variants represents the threshold above which an individual develops NTDs [109]. This study did not include analyses of regulatory variants that have been hypothesized to play an important role in the etiology of complex traits. It is of course possible that both models, rare alleles with large effects and rare alleles with minor effects, exist for severe NTDs. The omnigenic model was proposed to explain the missing heritability of complex traits. It assumes that any given disease phenotype can be directly affected by a modest number of core pathways and genes with specific roles in disease pathogenesis. However, a large number of interconnected regulatory networks and genes that are outside core pathways can affect the function of these core pathways. Since core genes only constitute a tiny fraction of all genes, most heritability comes from minor and indirect effects of hundreds or even thousands of genes expressed in the disease relevant cell [110].

When discussing the genetic components of NTDs, it is important to differentiate between severe NTDs from the milder closed forms that do not affect reproductive fitness. Gene burden studies in PCP signaling were either conducted in a cohort of open and closed NTDs without stratification by clinical type or only in open NTDs. The only two NGS studies of NTDs were conducted on the two severe open forms of MMC and anencephaly. While this review cannot speculate on the genetic architecture of milder forms of NTDs without more focused studies of larger cohorts, the fact that they do not affect reproductive fitness argues against a major role of rare alleles with large effects in their etiology.

To add complexity to the etiology of NTDs, a possible role that should not be excluded is for common variants of small effect size that could still contribute to the background liability, genotype-genotype interactions, genotype-environment interactions, and epigenetic effects. NTDs might indeed belong to a broad sense heritability model that takes into accounts all these genetic, environmental, and epigenetic factors [111].

## 6. Conclusions and Challenges

The evidence for an important role of PCP signaling in NTDs’ pathogenesis in vertebrate models is overwhelming. These models remain crucial for dissecting the molecular and cellular mechanisms by which PCP signaling mediates the process of CE and interacts with other developmental processes and pathways during neural tube formation. These models are also essential for the interpretation of the role of genetic variants identified in human studies and for the investigation of the role of gene-gene and gene-environment interactions in the NTDs’ etiology. In humans, the evidence has accumulated for an important contribution of PCP genes to NTDs’ pathogenesis. The majority of these variants were heterozygous, missense and incompletely penetrant, consistent with a complex etiology that implicates a large number of factors that together increase the risk of NTDs. As PCP mutations are not completely penetrant, it would still not be possible to offer an accurate recurrence risk after identifying a mutation in a PCP gene.

Despite intensive efforts, the etiology of NTDs remain a challenge and thousands of families are still affected resulting in a significant clinical, emotional and financial burden to the society. As genes and variants that are responsible for these devastating conditions are identified through NGS strategies, large collaborative efforts are needed to dissect this etiology and only then a better picture of the relative contribution of PCP signaling as well as other disease-relevant signaling pathways will emerge. In the more distant future, advances in genome technologies and in bioinformatics pipelines as well as the devotion of a large community of NTD researchers will provide affected families and clinicians with the tools for a personalized medicine in addition to better estimates of risk factors and appropriate prevention and counseling strategies.

## Figures and Tables

**Table 1:** List of rare coding PCP variants identified in neural tube defects (NTDs) and predicted to be damaging and/or affect protein function in NTDs.

Gene	NTD Cohort	Variant	Patient Description	Functional Validation	Reference
**PCP core genes**					
***ANKRD6***	391 Italians and 82 French Canadians (14 cranial, 246 MMC, and 213 closed spinal NTDs)	p.Pro548Leu p.Arg587Gln p.Arg632His	MC LipoMS and MC CA	p.Pro548Leu and p.Arg632His were proven to be partially loss of function in activation of PCP signaling and inhibition of canonical Wnt pathway	[95]
***CELSR1***	36 fetuses with CRS from US, France and England	p.Ala773Val p.Arg2438Gln p.Ser2964Leu p.Pro2983Ala	1 CRS 1 CRS 2 CRS 2 CRS	All 4 variants significantly affected frequency of membrane localisation	[90]
	391 Italians and 82 French Canadians (same as above)	p.Arg541Trp p.Val551Met p.Gln834X p.Arg.836Cys p.Val1008Leu p.Asp1401Gly p.Thr1443Pro p.Arg1456Gln p.Arg1526Trp p.Arg1835Cys p.Arg2121Cys p.Ser2190Leu p.Ala2228Val p.Arg2359Cys p.Ser2963Thr2966del	MMC LMC MMC Lipoma TFT MMC LMMC CA MMC MMC LMMC Lipoma Lipoma LMC CA	Not conducted	[87]
	192 American SB infants	p.Ala1023Gly p.Ile1124Met p.Thr1362Met p.Gly1410Arg Truncated protein (c.5050_5051 ins GT) p.Gly1825Ser Truncated protein (c.5719_5720 del TG) p.Gly2062Ser p.Arg2354Cys p.Arg2497Cys	All SB	Two truncated mutations disrupted CELSR1 membrane localization and its recruitment of Vangl2 to cell membrane.	[94]
	352 Chinese patients (26 CRS, 73EC, 64AN, 3Ex, 255 SB, 1 unknown)	p.Pro870Leu	CRS	p.Pro870Leu was a gain-of-function variant in zebrafish overexpression and rescue experiments. It increased both PCP and canonical Wnt signalling	[99]
	184 Chinese patients (36 AN,12 CRS, 32 EC, 2 Ex, 91 SB, 11 unknown) and 292 American patients (100% SB)	p.Arg714His p.Pro870Leu p.Thr875Ile p.Ala1019Ser p.Thr1086Met p.Arg1194His p.Gln1473Ter	AN, SB SB SB	Not conducted	[100]
	510 Chinese patients (125 AN, 232 SB, 46 EC, 79 AN & SB, 1 AN & EC, 15 SB & EC, 4 AN & SB & EC, 8 unknown)	p. Arg769Trp p.Arg1057Cys p.Gly1122Ser p.Gln2924His	SB SB AN SB	Not conducted	[101]
***CELSR2***	352 Chinese patients (same as above)	p.Ser628Gly p.Thr2026Met p.Arg2153Gly p.Arg2480Cys p.Arg2015Gly fs*22 p.Phe2397Lys fs*584	AN, SB SB EC SB CRS SB	p.Thr2026Met downregulated PCP signaling in a luciferase assay	[99,100]
	184 Chinese patients (same as above)	p.Ser628Gly p.Arg1990His p.Arg2015Gly fs*23 p.Thr2026His p.Arg2153Gly p.Arg2480Cys p.Arg2626Cys		Not conducted	[100]
***CELSR3***	352 Chinese patients (same as above)	p.Gly194Val p.Val446Met p.Gly1754Asp	1 AN, 1 SB CRS CRS	Not conducted	[99]
	184 Chinese patients (same as above)	p.Val446Met p.Ile1102Leu p.Arg1194His p.Arg1453Cys p.Gly1754Asp p.Gly1754Ser p.Val2584Gly p.Met2630Ile		Not conducted	[100]
***DVL2***	473 Italian and French Canadian patients (same as above)	p.Ala53Val p.Glu620X (c.1801_1802ins) p.Tyr667Cys	Lipoma CA and TC MMC	Not conducted	[91]
***DVL3***	184 Chinese patients (same as above)	p.Asp403Asn	SB	p.Asp403Asn disrupted DVL3 interaction with VANGL2, upregulated canonical Wnt signaling and down regulated non-PCP signaling	[100]
	510 Chinese patients (same as above)	p.Arg148Gln	AN	Not conducted	[101]
***FZD6***	473 Italian and French Canadian patients (same as above)	p.Cys615Ter p.Arg405Gln p.Arg511Cys p.Arg511His	MC MMC MMC CA	Not conducted	[89]
***PK1***	810 patients: 421 Italian (11 cranial, 211 open spinal including 208 MMC, 199 closed spinal) and 389 Americans (4 cranial, 325 MMC, 60 closed spinal)	p.Ile69Thr p.Asn81His p.Thr275Met p.Val550Met p.Arg682Cys p.Ser739Phe p.Asp771Asn	DM MMC MMC MMC MMC MMC CA	p.Ile69Thr, p.Asn81His, p.Thr275Met and p.Arg682Cys acted as hypermorphic alleles in inducing CE defects in an overexpression assay in zebrafish; p.Arg682Cys antagonized the CE phenotype induced by the wild-type *zpk1a* in a dominant fashion	[85]
***VANGL1***	137 Italian patients (80 MMC, 57 closed spinal) 7 French fetuses with CRS	p.Val239Ile p.Arg274Gln p.Met328Thr	CA MMC MMC, TC	p.Val239Ile abrogated interaction between VANGL1 and all three Dvl proteins; p.Val239Ile and p.Met328Thr failed to induce CE defects in overexpression assays and to rescue MO-induced CE defects in zebrafish	[81,104]
	673 patients: 284 Italians (11 cranial, 131 open spinal including 128 MMC, 142 closed spinal) and 389 Americans (4 cranial, 325 MMC, 60 closed spinal)	p.Ser83Ile p.Phe153Ser p.Arg181Gln p.Leu202Phe p.Ala404Ser	3 TFT and TC TFT and TC MMC MMC CA	Not conducted	[83]
	144 patients with open or closed NTDs from Slovakia, Romania and Germany	p.Gly205Arg p.Arg186His p.Arg173His	MMC TC and lipoma unknown	Not conducted	[88]
	53 Italian patients (9 MMC, 44 closed spinal)	p.Alal187Val p.Arg517His p.His350 His	LipoMS MMC Cephalocele	Not conducted	[97]
***VANGL2***	66 English patients (21 CRS, 24 SB, 21 AN)	A 7 bp duplication detected 30 nucleotides into intron six (IVS6+30)	CRS	Not conducted	[82]
	163 Han Chinese fetuses (16 AN, 63 CRS, 8 EC, 4 HPS, 14 iniencephaly, 58 SB)	p.Ser84Phe p.Arg353Cys p.Phe437Ser	HPS AN with SB AN	p.Phe437Ser completely abrogated interaction with Dvl; p.Arg353Cys diminished but did not abolish this interaction	[84]
	673 Italian and American patients (same as above)	p.Arg135Trp p.Arg177His p.Leu242Val p.Thr247Met p.Arg270His p.Arg482His	MMC DM 1MCS, 1MMC Lipoma Fibrolipoma CA and TC	Not conducted	[86]
**PCP mediators**					
***LRP6***	285 Italian patients (6 closed cranial, 153 MMC, 126 closed spinal)	p.Tyr306His p.Thr373Cys p.Val1386Leu p.Thr1541Cys	MMC MMC EC CA	p.Tyr306His, p.Thr373Cys and p.Val1386Leu acted as hypomorphic alleles in activating canonical Wnt pathway and inhibiting PCP signaling	[93]
	192 American SB infants	p.Ala3Val p.Tyr544Cys p.Pro1482Leu p.Arg1574Leu	SB	p.Tyr544Cys lost its ability to bind MESD and its membrane localization and acted as a hypomorhic allele in activating canonical Wnt signalling; p.Arg1574Leu acted as a hypermorphic allele in activating canonical Wnt signaling; p.Pro1482Leu lost its ability to inhibit PCP signaling	[96]
***PTK7***	473 Italian and French Canadian patients (same as above)	p.Ile121Met p.Val291Ile p.Pro345Leu p.Gly348Ser p.Pro545Arg	MMC LipoMS MC MMC MMC	p.Ile121Met, p.Pro345Leu and p.Pro545Arg could act in a hypomorphic manner; p.Gly348Ser acted in a hypermorphic manner in overexpression assays in zebrafish;p.Pro545Arg affected stability of protein	[98,105]
	343 Chinese patients (70 AN, 19 CRS, 80 EC, 3 EX, 170 SB, 1 unknown) and 192 American SB infants	p.Asn128Ser p.Thr186Met p.Ala560Thr p.Arg630Ser p.Pro706Arg p.Tyr725Phe p.Gly765Arg p.Val775Met p.Arg790Leu	EX SB SB SB 2 AN, CRS, 2 SB SB 2 SB SB 2 SB, EC, AN	p.Arg630Ser affected protein stability and increased interaction with Dvl2; p.Thr186Met decreased PTK7 interactions with Dvl2	[102]
	510 Chinese patients (same as above)	p.Pro642Arg	SB	Not conducted	[101]
***SCRIB1***	52 fetuses with CRS (same as above)	p.Arg1535Gln	CRS	p.Arg1535Gln affected membrane localization of Scrib1	[90]
	192 American SB infants	p.Ala366Thr p.Thr552Met p.Pro1043Leu p.Pro1332Leu p.Leu1520Arg	SB	p.Pro1043Leu, p.Pro1332Leu and p.Leu1520Arg significantly affected protein membrane localization	[92]
	473 Italian and French Canadian patients (same as above)	p.Gly263Ser p.Pro649His p.Gln808His p.Arg1150Gln p.Thr1422Met	MMC CA MMC VS MMC	P.Gly263Ser and p.Gln808His significantly affected the subcellular localization of SCRIB1 and failed in rescuing the localization defect of Par-3 and Vangl1 caused by knockdown of *Scrib1*; P.Gln808His and p.Arg1150Gln abolished the interaction of Scrib1 with Vangl2.	[68]
	510 Chinese patients (same as above)	p.Lys618Arg p.Gly644Val p.Arg1044Gln p.Gly1108Glu	SB SB AN SB	Not conducted	[101]

AN, anencephaly; CA, caudal agenesis; CRS, craniorachischisis; DM, diastematomyelia; EC, encephalocele; Ex, exencephaly; HPS, holoprosencephaly; LipoMS, lipomyeloschisis; LMC, lipomyelocele; LMMC, lipomyelomeningocele; MC, meningocele; MCS, myelocystocele; MMC, myelomeningocele; NTD, neural tube defect; PCP, planar cell polarity; SB, spina bifida; TC, tethered cord; TFT, tight filum terminale; VS, vertebral schisis.
